# Filamentous structure of the CotVW complex, the crust proteins of the *Bacillus subtilis* endospore

**DOI:** 10.1016/j.jbc.2025.110714

**Published:** 2025-09-11

**Authors:** Eunbyul Jo, Doyeon Kim, Yeongjin Baek, Migak Park, Hyojeong Lee, Nam-Chul Ha

**Affiliations:** 1 Research Institute of Agriculture and Life Sciences, Department of Agricultural Biotechnology, CALS, Seoul National University, Seoul, Republic of Korea; 2 Center for Food and Bioconvergence, Department of Agricultural Biotechnology, Interdisciplinary Programs in Agricultural Genomics, CALS, Seoul National University, Seoul, Republic of Korea

**Keywords:** CotVW, helical filament, *Bacillus* spore, crust, cryo-EM structure, pH-dependent, interaction, sporulation, germination

## Abstract

The endospores of *Bacillus subtilis* are encased in a multilayered protective structure comprising core, cortex, inner and outer coats, and an outermost crust. Among the proteins required for crust formation, CotV and CotW are unique to *B. subtilis* and are hypothesized to be instrumental in maintaining spore surface integrity. However, their structural organization and functional mechanisms remain unclear. This study determined the cryo-EM structure of the CotVW complex and revealed its filamentous helical architecture. Structural analysis showed that CotVW possesses a negatively charged surface that enables pH-dependent binding interactions. Specifically, at pH 6.0, CotVW engages in electrostatic interactions with histidine and positively charged residues, suggesting a potential regulatory mechanism influenced by the environmental pH. Our results elucidate the molecular basis of CotVW function in *B. subtilis* spore crust formation, highlighting its role in spore surface organization. This study advances our understanding of the spore coat architecture and may inform future research on bacterial spore resilience and structural adaptation.

*Bacillus subtilis* is a widely used bacterium with applications in industry, agriculture, and medicine, owing to its well-characterized genetic background and safety profile (1). Its endospores, renowned for their exceptional stability and resistance, play a key role in processes such as food fermentation (2). *Bacillus* endospores possess a multilayered structure comprising distinct layers with specialized functions: core, cortex, inner and outer coat, and crust (3, 4, 5, 6). Each layer is assembled through the coordinated interaction of various coat proteins, with key morphogenetic proteins driving this process (7). Morphogenetic proteins of the basement layer contribute to spore cortex formation, coat assembly, anchoring of the coat to the spore surface, and spore encasement (8, 9). The outer coat, which forms the external spore coat, relies on CotE, an essential morphogenetic protein (10, 11). The flexible, mesh-like structure of CotE facilitates the assembly and structural stability of the outer coat while serving as a foundation for interactions between the outer coat and crust (12, 13, 14).

The crust is the outermost layer of *B. subtilis* spores (8, 15). CotY and CotZ are essential morphogenetic proteins that assemble the crust layer in a mutually dependent manner (5, 15). Both proteins are enriched in cysteine residues and cooperatively form hexagonal crystalline arrays at the spore surface (13). Especially, CotY contains a C-terminal region with positively charged residues, including multiple histidine residues, which are predicted by AlphaFold 3 to form a flexible loop (16). CotY initiates layer formation at the spore poles, whereas CotZ anchors these layers centrally to ensure cohesive assembly and structural integrity (3, 17).

CotV and CotW, found only in *B. subtilis* clans, are spore coat proteins belonging to a distinct but equally significant superfamily within the crust (3). Although these proteins are hypothesized to be instrumental in the structural organization, extension, and overall architecture of the crust, their specific functions and interactions remain unclear (3, 18, 19). For instance, CotV influences the spatial distribution and continuity of the crust across the spore surface, whereas CotW contributes to structural cohesion and stability. CotW appears to be evenly distributed across the spore surface and may interact with outer coat proteins, suggesting a potential integrative function. CotV is likely dependent on CotW for its localization and function, with evidence indicating the formation of filamentous complexes between the two proteins (3, 19, 20).

Despite these hypotheses, the precise molecular mechanisms governing CotV and CotW function, their structural characteristics, and their exact spatial arrangements in the crust remain largely unknown. CotV and CotW proteins may work cooperatively to reinforce the crust. A low-resolution negative-stain electron microscopy (negative-stain EM) study indicated that the CotV and CotW complex (referred to as CotVW in this study) form a linear, rope-like polymer (13). However, detailed structural and mechanistic insights are still lacking. In the present study, we determined the cryo-EM structure of the CotVW complex and suggested its functional role in crust assembly. These findings offer valuable insights into the molecular mechanisms governing spore crust formation and contribute to a deeper understanding of the structural basis of spore durability.

## Results

### Purification and characterization of the filamentous CotVW complex

The *cotV* and *cotW* genes from the *B. subtilis* strain 168 were cloned into the pET-DUET vector to coexpress the two proteins in the *Escherichia coli* cytoplasm. A 6xHis-tag was introduced exclusively at the N terminus of CotV, enabling affinity purification of the CotVW complex using Ni-NTA chromatography (Fig. S1, *A* and *B*). Negative-stain EM revealed that the purified CotVW complex assembled into distinct filamentous structures, consistent with those reported previously (13), although substantially shorter in length (Fig. S1*C*). We suspect that longer CotVW filaments were partially retained by the bottom frit of the Ni-NTA column, resulting in the selective loss of high-molecular weight species during elution.

**Figure 1:**
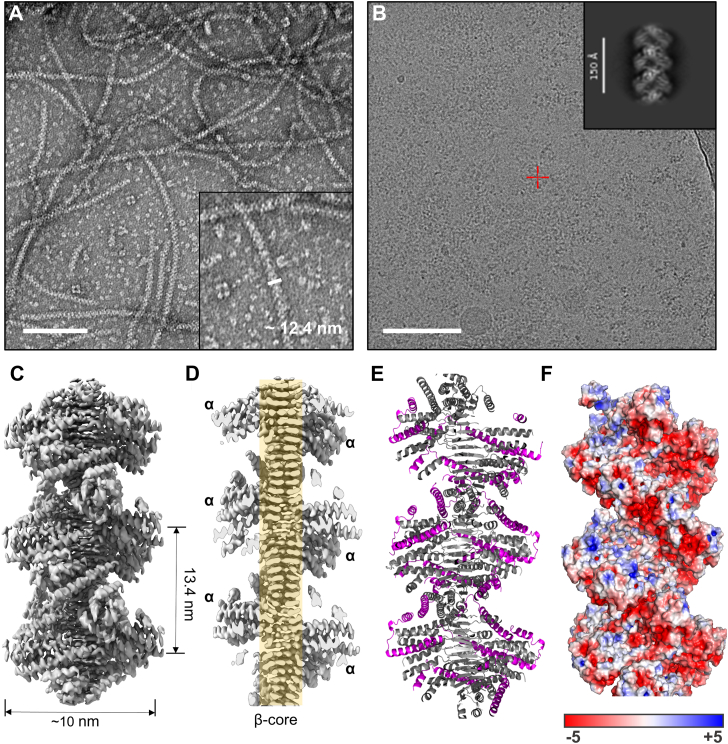
**EM structures of CotVW filaments.***A*, negative-stain EM images of CotVW filaments from the cell lysate of *Escherichia coli* coexpressing 6xHis-CotV and CotW (6xHis tagged CotVW complex). The width of the filaments was measured at 12.4 nm in the image. The *white* scale bar represents 50 nm. *B*, cryo-EM images of 6xHis CotVW filaments. Two-dimensional class averaged images are at the *top right* corner. The *white* scale bar represents 50 nm. *C*, 3D model from the cryo-EM images built with helical symmetry. The apparent helical pitch is 13.4 nm and width is ∼10 nm. *D*, cross-section of Fig. 1*C* at the center along the filament axis. The central β core (β) is shaded in *yellow*. The α-helical domains (α) are surrounded by the β core. *E*, CotW location in the CotV (*gray*) and CotW (*magenta*) complex. *F*, electrostatic surface built by PyMol (29). The electrostatic spectrum is displayed ranging from *red* (−5) to *blue* (+5).

To better preserve filament integrity, we conducted negative-stain EM using the column-loading sample (pre-Ni-NTA), which retained a greater abundance of long filaments, consistent with previous observations (13) (Fig. 1*A*). These filaments exhibited clear helical organization, with globular subunits arranged in a repeating pattern along the filament axis, indicative of an ordered supramolecular assembly. In addition, the filaments displayed slight curvature, suggesting an intrinsic degree of structural flexibility.

### Double-helical structure of the CotVW filament

The raw cryo-EM data of the CotVW filaments (Fig. 1*B*) revealed filamentous structures similar to those observed in negative-stain EM. In total, 4.742 raw images were processed using CryoSPARC (21). The two-dimensional (2D) class-averaged images displayed filamentous structures helically wrapped by globular domains (Fig. 1*B*). The 3D model generated after applying a helical symmetry operation (Fig. S2) produced a high-resolution cryo-EM map at 3.32 Å resolution (Figs. 1*C* and S2). The filament had a diameter of approximately 10 nm with a helical pitch of 13.4 nm measured along the grooves (Fig. 1*C*). The cryo-EM map distinctly revealed the central β-strand core (β core) and surrounding α-helical domains along the filament axis in a cross-sectional view (Fig. 1*D*).

We constructed an atomic model for the cryo-EM map using the artificial intelligence driven protein model-building tool, ModelAngelo (22). The native CotV and CotW proteins from *B. subtilis* strain 168 consist of 129 and 105 residues, respectively. A N-terminal 6xHis-tag and C-terminal additional tyrosine residue were linked to CotV to facilitate purification. The final atomic model includes residues 1 to 125 of CotV and 18 to 98 of CotW. The additional residues are unlikely to affect the overall structure, and were not resolved in the cryo-EM map.

The final model revealed 1:1 stoichiometry of CotV and CotW. Further analysis showed that the central β-core was composed exclusively of CotV, whereas the peripheral α-helical domains consisted of both CotV and CotW (Fig. 1*E*). The filament surfaces were predominantly hydrophilic, and electrostatic analysis indicated enrichment of acidic residues along the grooves of the double helix (Fig. 1*F*). This structural feature suggests that CotVW filaments can bind positively charged peptides or proteins *via* interactions with their groove surfaces.

### Intermeshing β-strand interaction between the monomers

**Figure 2:**
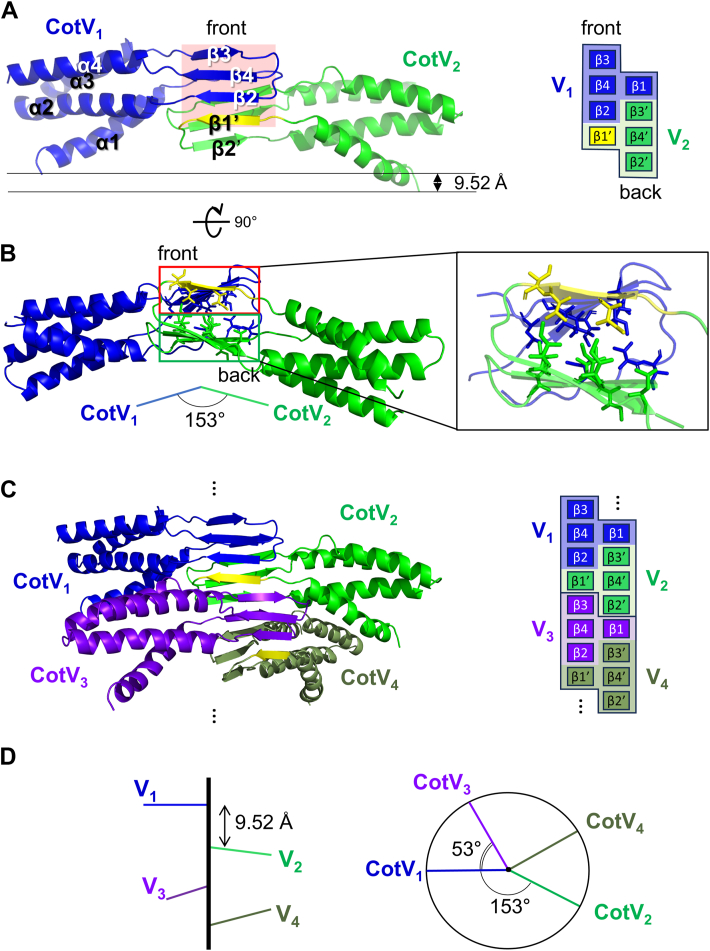
**Structural organization of CotV filaments and intersubunit interactions.***A*, two directly neighboring subunits of CotV (CotV_1_ in *blue* and CotV_2_ in *green*) related by helical symmetry with a rise of 9.52 Å and twist of 153.09°. The β-sheet on the front face is highlighted in *red* and consists of β3, β4, and β2 from subunit CotV_1_ (*blue*) and β1′ from subunit CotV_2_ (*yellow*, within the *green* subunit). The schematic representation in the *rightpanel* depicts the front and back β-sheets. β1–4 from subunit CotV_1_ (V_1_) are shown in *blue* and *blue* shading, and β1′–4′ from subunit CotV_2_ (V_2_) are in *green* with *green* shading, except for β1′, which is highlighted in *yellow*. The vertical rise between adjacent subunits along the filament axis is 9.52 Å. *B*, end-on view of Fig. 2*A*. The hydrophobic interactions between the front β-sheet (*red* box) and back β-sheet (*green* box) are represented with *ribbons*. The angle between the two directly adjacent subunits is 153° and corresponds to the helical twist of the filament. *C*, intermeshing β-strands formed by four consecutive subunits (CotV_1_, CotV_2_, CotV_3_, and CotV_4_) along the filament axis, each represented in a different color. The angle between directly neighboring subunits (*e.g.*, CotV_1_ and CotV_2_) is obtuse (153°), whereas the angle between every other subunit (*e.g.*, CotV_1_ and CotV_3_) is 53°. Consequently, subunits CotV_1_ and CotV_3_ are positioned in closer proximity compared to CotV_1_ and CotV_2_. The schematic arrangements are shown on the *right*: each CotV subunit (V_1_–V_4_) is represented by a line in the same color code. *D*, schematic representation of the helical rise and twist angles between four consecutive CotV subunits (V1–V4) along the filament axis. Each subunit is represented as a colored line consistent with *panels C*. The vertical distance between adjacent subunits corresponds to a helical rise of 9.52 Å, and the angular displacement (helical twist) between them is 153°. The angle between every other subunit (*e.g.*, V1 and V3) is 53.

The central β core was primarily composed of two long β-sheets facing each other (Fig. 2*A*, front and back β-sheets). The CotV monomer consisted of four α helices in the α-helical domain and four β-strands in the β core (Fig. 2*A*). Secondary structure analysis showed that the alternating α-helices and β-strands were α1-β1-β2-α2-α3-β3-β4-α4. The four α-helices formed a four-helical bundle structure, and the β-strands formed two central β-sheets: β2–4 for the front sheet and β1 for the back sheet (Fig. 2*A*). These four β-strands were further organized into a single β-strand in a sheet and β-sheet consisting of three β-strands (β3, β4, and β2 in the front sheet, and β1 in the back sheet). Notably, β1 aligned with the middle β4 on the opposite face at the same level within the β core. Consequently, β1 protruded onto the β3, β4, and β2 sheets, creating a T-block structure reminiscent of a Tetris game piece (blue shaded region in Fig. 2*A*, right panel).

One CotV monomer (labeled as CotV_1_ in Fig. 2*A*, blue) interacts with β-strands from adjacent CotV subunits along the helical axis. Specifically, β3 and β4 of CotV_1_ intercalate with β2′ and β1′ of CotV_2_, forming a cross-unit β-sheet interface through the gaps between T-blocks (shaded regions in Fig. 2*A* right). This stacking arrangement creates an extended β-sheet configuration where β-strands from adjacent monomers are offset along the filament axis, unlike canonical amyloid filaments in which all β-strands are aligned at the same height within the β-core. In addition to the interstrand hydrogen bonds, strong hydrophobic interactions are also observed between the opposing β-sheets (Fig. 2*B*), further stabilizing the filament core.

Along the helical axis, CotV monomers stack with a helical rise of 9.52 Å and a twist of 153.09°, forming a continuous spiral of subunits (Fig. 2, *C* and *D*). This arrangement gives rise to two helically neighboring sets of CotV subunits—odd-numbered (CotV_1_, CotV_3_, CotV_5_, …) and even-numbered (CotV_2_, CotV_4_, CotV_6_, …) monomers—each forming a distinct thread within the filament. Accordingly, the CotV filament assembled into a two-threaded (grooved) helical structure, resembling a double helix. However, unlike the antiparallel architecture of DNA, where two complementary strands run in opposite directions, the CotV filament adopted a parallel double-helical arrangement, with both threads aligned in the same axial direction (Fig. S3). This parallel arrangement is a key structural feature that distinguishes CotV filaments from classical helical biomolecules such as DNA. The repeating unit of this double-helical filament comprises seven pairs of odd- and even-numbered CotV monomers, resulting in a helical offset of −376° rotation (calculated as 54° × 7) with a helical pitch of 13.4 nm per one full repeat (Fig. S3).

### CotW, the connecting α helices between the CotV protomers

**Figure 3:**
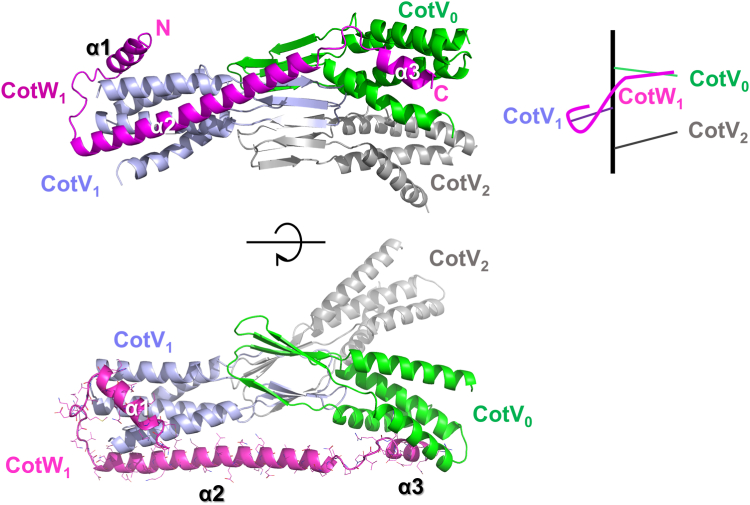
**Structural organization of CotV and CotW.** Orthogonal views (*front* view, *top*; *top* view, and *bottom*) of the CotVW filament highlight CotW (CotW_1_; *magenta*) from CotV_0_ (*green*), CotV_1_ (*pale violet*), CotV_2_ (*gray*). CotW_1_ spans both CotV_0_ and CotV_1_, bridging adjacent subunits. Each secondary structural element of CotW_1_ is labeled.

The observed portion of the CotW monomer (residues 18–98) in the electron density map consisted of three α-helices and their associated loops. CotW formed the α-helical domains of two adjacent CotV molecules. α1 (residues 21–32) and α2 (residues 44–82) of CotW encircled the α-helical domain of CotV, contributing to its structural integrity. Notably, α3 (residues 90–97) extended toward an adjacent CotV subunit through a linking loop, thereby facilitating intersubunit connectivity within the filament (Fig. 3*A*). This structural arrangement suggests that CotW plays a critical role in stabilizing CotV filaments by bridging the α-helical domains of adjacent units and shielding the hydrophobic surfaces of the filament from solvent exposure.

### Disruption of the CotVW filament by Ca and Ca-DPA (calcium-dipicolinic acid)

To evaluate the structural stability of the CotVW filament against detergents, a strong acid, strong base, 1% SDS (boiled), 1 M HCl, or 1 M NaOH were added to the CotVW filament sample. The filaments were completely or mostly dissembled in 1 M HCl and 1 M NaOH, as shown in the negative-stain EM images. Either heat at 80 °C or sonication treatments preserved the long filamentous structure without observable structural changes, demonstrating CotVW’s remarkable heat resistance, consistent with the thermal durability of endospores (Fig. S4).

**Figure 4:**
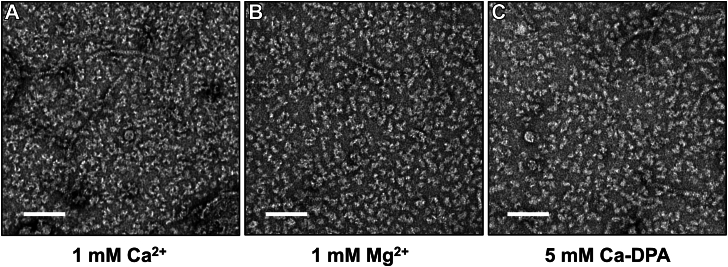
**Disruption of nontagged CotVW filaments by Ca^2+^ and Ca-DPA.***A–C*, negative-stain EM images of the nontagged CotVW filaments in 20 mM Tris buffer (pH 8.0) containing 150 mM NaCl and indicated additives (1 mM CaCl_2_, 1 mM MgCl_2_, and 5 mM Ca-DPA). The *white* scale bar represents 100 nm. Additional conditions (*e.g.*, Ni^2+^, Mn^2+^) are shown in Fig. S5. Ca-DPA, calcium-dipicolinic acid.

Next, we exposed CotVW filaments to various divalent metal ions at a concentration of 1 mM, which disrupted the CotVW filaments into small fragments (Figs. 4, *A*, *B* and S5). Under negative-stain EM, the otherwise stable CotVW filaments, maintained in 20 mM Tris buffer (pH 8.0) with 150 mM NaCl, became fragmented upon the addition of CaCl_2_, MgCl_2_, or MnCl_2_. Treatment with calcium-dipicolinic acid induced the same transition into small filamentous fragments in the dose-dependent manner (Figs. 4, *A, B* and S5). This disruption implies that divalent metal ions induce conformational or structural changes in CotVW filaments during the sporulation or germination steps.

### Comparison of 6xHis-tagged CotVW and nontagged CotVW filaments

**Figure 5:**
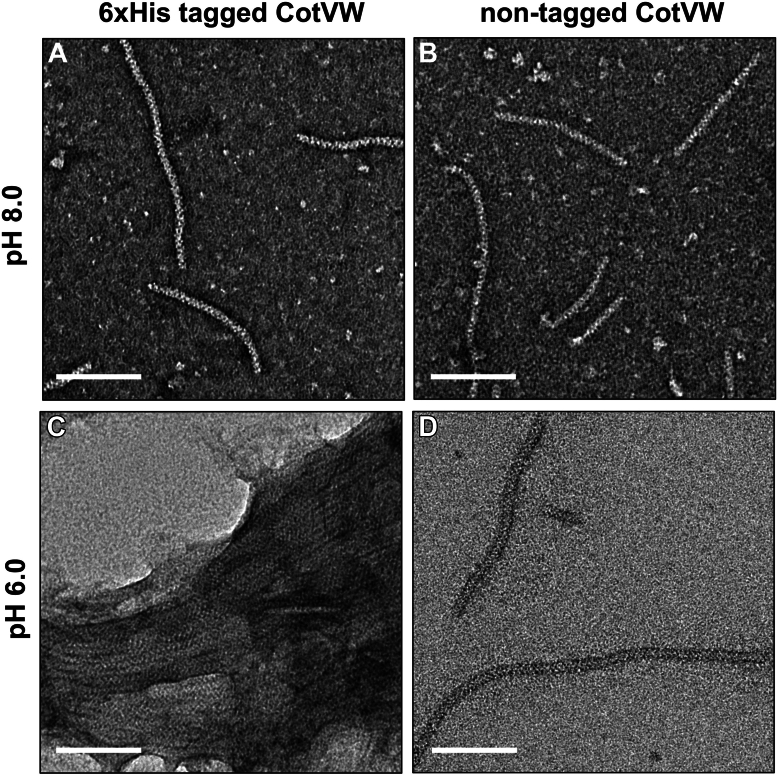
**Comparison of 6×His-tagged and nontagged CotVW filaments under different pH conditions.** Negative-stain EM analysis of 6×His-tagged CotVW (*A*, *C*) and nontagged CotVW filaments (*B*, *D*) at pH 8.0 (*A*, *B*) and pH 6.0 (*C*, *D*). The *white* scale bar represents 50 nm.

To compare the cryo-EM structures of the 6xHis-tagged CotVW analyzed in this study, nontagged CotVW filaments were purified using the same protocol without applying the Ni-NTA affinity chromatography. Negative-stain EM analysis of the nontagged filaments revealed structural features indistinguishable from those of the 6×His-tagged filaments at pH 8.0 (Fig. 5, *A* and *B*). This result confirmed that the cryo-EM structure of the 6xHis-tagged CotVW filaments accurately represented the native conformation of CotVW.

Further analyses were conducted to investigate the structural organization of the 6×His-tagged and untagged CotVW filaments under acidic conditions (pH 6.0). Negative-stain EM imaging revealed that the 6xHis-tagged CotVW filaments underwent lateral association, forming higher-order filament bundles, whereas the nontagged filaments remained as discrete individual filaments, consistent with their morphology at pH 8.0 (Fig. 5, *C* and *D*). These findings suggest that the lateral bundling of CotVW filaments at pH 6.0 is driven by interfilament interactions mediated by the histidine-rich 6xHis tag, likely resulting from increased protonation of histidine residues under acidic conditions, leading to enhanced electrostatic interactions.

**Figure 6:**
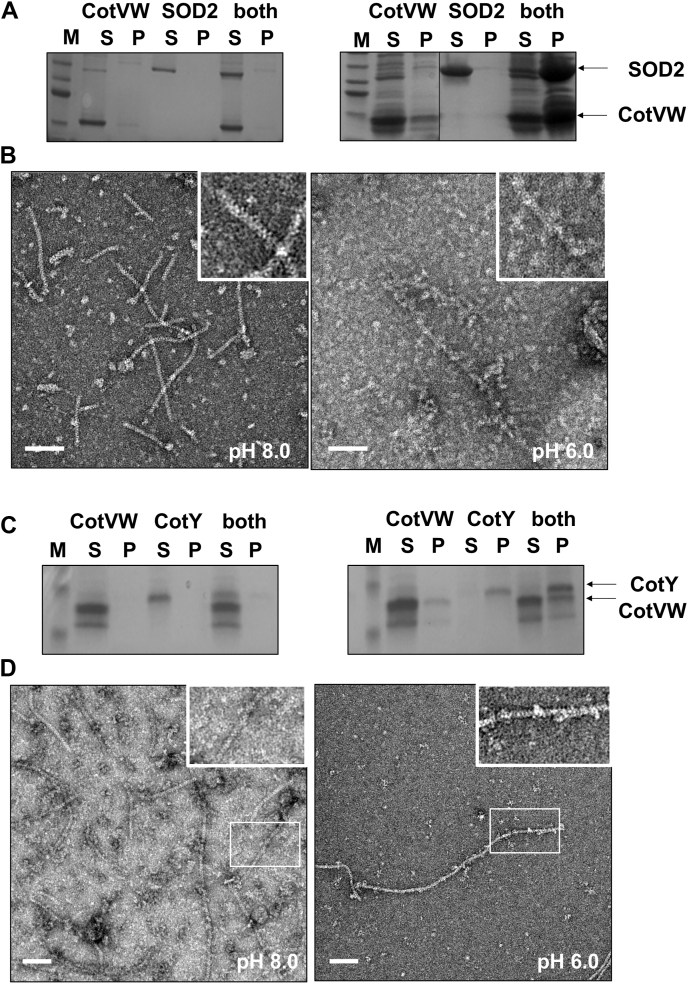
**pH-dependent molecular interactions between nontagged CotVW filaments and histidine-rich proteins (6×His-SOD2 and CotY).***A*, SDS-PAGE analysis of CotVW, 6×His-tagged SOD2 (SOD), and their mixture after spin-down centrifugation to separate the supernatant (S) and precipitate (P) fractions at pH 8.0 (*left*) and pH 6.0 (*right*). The *white* scale bar represents 100 nm. *B*, negative-stain EM images of samples containing both CotVW and 6×His-tagged SOD2 at pH 8.0 (*left*) and pH 6.0 (*right*). The *white* scale bar represents 100 nm. *C*, SDS-PAGE analysis of CotVW, CotY, and their mixture after spin-down centrifugation to separate the supernatant (S) and precipitate (P) fractions at pH 8.0 (*left*) and pH 6.0 (*right*). The *white* scale bar represents 100 nm. *D*, negative-stain EM images of samples containing both CotVW and CotY at pH 8.0 (*left*) and pH 6.0 (*right*). The *white* scale bar represents 100 nm. SOD2, superoxide dismutase 2.

To further investigate whether CotVW filaments specifically recognize histidine residues and bind *via* electrostatic interactions, binding assays were performed using nontagged CotVW filaments and 6xHis-tagged protein. In this study, we used 6xHis-tagged superoxide dismutase 2 (SOD2), a tetrameric protein, as a surrogate for histidine-rich proteins. Notably, the soluble 6xHis-tagged SOD2 protein coprecipitated with the nontagged CotVW filaments at pH 6.0, but not at pH 8.0 (Fig. 5*A*). Consistently, negative-stain EM analysis at pH 6.0 revealed complex formation with irregularly branched filaments, whereas no additional density was observed on the characteristic helical features of CotVW filaments at pH 8.0 (Fig. 6*B*). This finding is consistent with the previously observed pH-dependent bundling of 6xHis-tagged CotVW filaments and highlights the critical role of histidine clusters in facilitating binding under acidic conditions. These results strongly suggest that the protonation of histidine residues at acidic pH enhances electrostatic interactions with negatively charged regions on CotVW filaments, thereby promoting binding and inducing structural alterations.

### Electrostatic binding of CotY to the CotVW filament

CotY, a major crust protein in *B. subtilis*, forms hexamers that assemble into a 2D crystalline lattice composed of multiple hexameric units (13). Notably, the C-terminal region of CotY contained a histidine-rich sequence with two adjacent basic residues (KKHHHNG), which was predicted to adopt an exposed α-helical conformation according to AlphaFold 3 (Fig. S7) (23). To investigate the role of this histidine-rich region in protein interactions, we performed coprecipitation assays at pH 6.0, similar to those conducted with His-tagged SOD2. SDS-PAGE analysis revealed that CotVW filaments coprecipitated with CotY at pH 6.0 (Fig. 6*C*, right). Negative-stain EM of the precipitates further showed that hexameric CotY molecules were bound to the surface of CotVW filaments under acidic conditions (Fig. 6*D*, right). In contrast, at pH 8.0, no coprecipitation was observed, and negative-stain EM analysis showed no evidence of CotY binding to CotVW filaments (Fig. 6, *C* and *D*, left). These findings suggest that the C-terminal histidine-rich region of CotY hexamers mediates pH-dependent interactions with CotVW, thereby promoting protein association under acidic conditions.

## Discussion

In this study, we elucidated the high-resolution cryo-EM structure of the CotVW complex, which revealed a distinctive double-threaded helical screw-like architecture. This double-threaded architecture may contribute to the mechanical robustness or assembly dynamics of CotV filaments, with the alternating registration of odd and even subunits playing a potential role in guiding filament polarity or interaction with other crust components. Structural integrity appears to be predominantly conferred by the densely packed β core of CotV, whereas CotW contributed to stabilizing α-helices that wrap around this core and connected adjacent CotV subunits. CotW likely plays a dual role: reinforcing the filament architecture and acting as an amphipathic linker to the outer coat, as previously proposed (3, 19). The vertical stacking of CotV β-strands along the central axis, stabilized by intermeshing interactions, likely imparted both mechanical strength and a degree of conformational flexibility. These structural features may explain the remarkable resistance of CotVW filaments to heat and shear stress, underscoring their critical role in enhancing the mechanical robustness of the spore crust. A prominent feature of the filament is its highly negatively charged surface, especially along the grooves, which may contribute to specific electrostatic interactions with other coat proteins or ions during the crust layer assembly.

During *B. subtilis* sporulation, spore cores experience acidic pH conditions: the dormant spore core typically has a pH of ∼6.0 (24). We found that nontagged CotVW filaments exhibited specific interactions with 6×His-tagged proteins and the major crust component, CotY, at an acidic pH (pH 6.0). This pH-dependent association was likely mediated by the histidine-rich flexible C-terminal region of CotY, suggesting that CotW bridges the interactions between the CotVW filament and CotY during sporulation.

Our findings support the proposed role of CotVW as a molecular scaffold for CotY deposition. CotV was previously localized to the cap region of the forespore alongside CotY, and has been implicated in the initiation of CotY assembly (3, 19). Here, we provide direct evidence of CotVW binding to CotY hexamers under acidic conditions, which is consistent with the environment during sporulation. The presence of multivalent CotY-binding sites along the CotVW filament suggests that CotVW filaments nucleate and organize CotY hexamers into a crystalline-like lattice at the forespore cap, promoting the spatially ordered construction of the crust layer.

This study provides a comprehensive structural and functional characterization of the CotVW complex and its role in the architecture and regulation of the spore crust. By resolving the filamentous organization and interaction with CotY, we highlighted the central mechanism by which structural and environmental factors converge to control spore coat formation and germination. These findings offer valuable insights into endospore biology and have potential applications in designing spore-based systems with tailored mechanical properties. In particular, the manipulation of CotVW-CotY interactions could inform strategies for enhancing probiotic stability, optimizing fermentation processes, and controlling spore persistence in industrial and clinical contexts.

## Experimental procedures

### Plasmid construction and expression of 6xHis-tagged and nontagged CotVW

The genes encoding CotV and CotW from *B. subtilis* 168, each appended with a C-terminal tyrosine to facilitate purification, were synthesized and inserted into the pET-DUET-1 vector for expression in *E. coli* (25). The CotV and CotW genes from *B. subtilis* 168 were synthesized using Gene Synthesis Services (Gene Universal) with codon optimization for *E. coli*. The synthesized CotVW genes were inserted into the BamHI and HindIII sites (for CotV) and the NdeI and XhoI sites (for CotW) of the pET-DUET vector to construct the recombinant pET-DUET-CotVW vector. The vector was designed to carry a 6xHis-tag at the N terminus of the CotV for ease of purification. For the nontagged construct, the same vector backbone was used, but the CotV gene was cloned into the NcoI and HindIII sites to exclude the N-terminal 6xHis-tag. The resulting expression vectors were transfected into *E. coli* BL21(DE3) cells (Merck). The transformed cells were cultured in 1.5 L of LB medium supplemented with 50 μg/ml ampicillin at 37 °C until reaching an OD_600_ of 0.6. Protein expression was then induced by adding 0.5 mM isopropyl-β-D-thiogalactoside (IPTG) to the culture medium, followed by an additional 6 h of incubation at 30 °C. The cells were harvested by centrifugation at 5500*g* for subsequent purification.

### Plasmid construction and protein expression of CotY

The *cotY* gene from *B. subtilis* 168 was codon-optimized for *E. coli* and synthesized by Gene Synthesis Services (Gene Universal and Bionics). Each gene was inserted between the NcoI and XhoI sites of the pET-28a vector. The recombinant vectors were transfected into *E. coli* BL21(DE3) cells and cultured in LB medium with 50 μg/ml kanamycin at 37 °C until *A*_600_ reached 0.6. Protein expression was induced with 0.5 mM IPTG, followed by 6 h of incubation at 30 °C. The cells were harvested by centrifugation at 5500*g* for further purification.

### Purification of the 6xHis-tagged and nontagged CotVW complex

The harvested *E. coli* cells expressing 6xHis-tagged and nontagged CotVW proteins were resuspended in 50 ml lysis buffer containing 20 mM Tris–HCl (pH 8.0) and 150 mM NaCl. The resuspended cells were disrupted by sonication at 70% power, and the resulting cell debris was removed by centrifugation at 20,000*g* for 30 min at 4 °C. No additional membrane-based chromatographic steps were performed to preserve the extended length of the non-tagged CotVW filament. Instead, the clarified supernatant obtained after removing cell debris was directly used for further studies.

To further purify the 6xHis-tagged CotVW complex, the supernatant was then applied to a Ni-NTA agarose resin (Qiagen) equilibrated with lysis buffer. After incubation at 4 °C for 1 h with gentle rotation, the resin was washed with buffer containing 20 mM imidazole to remove nonspecifically bound proteins. The His-tagged CotVW complex was eluted with buffer containing 250 mM imidazole and subsequently analyzed by SDS-PAGE and negative-stain EM.

### Purification of CotY

The harvested *E. coli* cells expressing CotY proteins were resuspended in 50 ml lysis buffer containing 20 mM Tris–HCl (pH 8.0) and 150 mM NaCl. The resuspended cells were disrupted using a French press (Constant Systems) at 23 kpsi and the resulting cell debris was removed by centrifugation at 20,000*g* for 30 min at 4 °C. The cell lysate was incubated with Ni-NTA agarose resin (Qiagen) with gentle rolling on a column (GE Healthcare). The resin was washed with 20 mM imidazole, and the protein was eluted using 250 mM imidazole. To remove the imidazole and exchange the buffer, the eluted protein was desalted using a HiTrap desalting column (5 ml; Cytiva). The desalting buffer was pH-dependent, with 20 mM Tris–HCl and 150 mM NaCl at pH 8.0, identical to the lysis buffer, and 20 mM Mes-NaOH and 150 mM NaCl at pH 6.0. Purified CotY was used for interaction assays with the CotVW complex.

### Negative-stain EM images of coat proteins

For negative staining, the CotVW complex samples were serially diluted 10-fold for imaging as individual complexes. CotY macromolecules were used without dilution for imaging purposes. When preparing the CotVW-CotY mixture, the concentration of each component was adjusted using Bradford assays to ensure proper stoichiometric balance. The prepared mixture was either used without further dilution or diluted 10-fold as needed for sample preparation. A 10 μl aliquot of each sample was deposited onto glow-discharged, 400-mesh carbon-coated copper grids (Electron Microscopy Sciences). The proteins adsorbed onto the grid surfaces were negatively stained with a 1% uranyl acetate solution and allowed to air-dry at room temperature under controlled conditions. Negative-stain EM imaging was conducted using a 120 kV Tecnai G2 Spirit TWIN transmission electron microscope (FEI, CMCI at Seoul National University) equipped with a Rio 4 CMOS camera (Gatan). Micrographs were captured at magnifications of 2400× and 21000× to ensure detailed visualization of both the individual macromolecules and mixed complex.

### Cryo-EM images of the CotVW complex

The purified CotVW complex, obtained as a 100-fold diluted supernatant, was used as the base sample. This solution was further diluted to approximately 0.8x its original concentration. Aliquots of 4 μl were deposited onto glow-discharged Q 1.2/1.3 Cu 300 mesh grids (Electron Microscopy Sciences). The grid surfaces were oxidized using the oxidizing agent ammonium persulfate, with cellophane covering the grid to generate a copper oxide spike grid (26). Glow discharge was performed at a current of 15 mA for 60 s to optimize the grid hydrophilicity. The sample was vitrified using a Vitrobot Mark IV system (Thermo Fisher Scientific Inc) with a blotting time of 5 s and blot force set to 0 under chamber conditions maintained at 4 °C and 100% humidity. Grid screening was performed using a 200 kV Glacios cryo-EM microscope (Thermo Fisher Scientific) to assess ice thickness, protein concentration, and filament visibility. Therefore, a grid with optimal ice and sample quality was selected for imaging. High-resolution cryo-EM data were acquired using a 300 kV Krios G4 microscope (Thermo Fisher Scientific). Images were recorded with a Falcon four detector in counting mode. The data collection parameters included a nominal magnification of 96,000× , which yielded a pixel size of 0.81 Å. The total exposure time was 8.28 s, fractionated into 24 frames (0.345 s per frame), with a total dose of 50 e/Å^2^ and dose per fraction of 2.08 e/Å^2^. The C2 lens aperture was set to 70 μm, and the objective lens aperture was 100 μm (Table S1).

### Cryo-EM processing of the CotVW complex

The cryo-EM image dataset obtained using the Krios microscope was processed using the cryo-EM software package CryoSPARC (21). We used an automatic filament-picking tool (Filament Tracer) and performed particle extraction (450 pixels) to collect filament particles. A total of 602,002 particles were successfully subjected to three cycles of 2D classification, as 2D model templates and class averages. The resulting 2D class averages were used as templates for the second round of automatic filament selection. Following template-based particle selection, particle extraction (250 pixels) was performed to improve the resolution. Ultimately, 137,931 particles were selected and subjected to 3D reconstruction *via* helical refinement.

During the initial stage of helical reconstruction, we utilized a helical symmetry search tool to identify optimal parameters for the helical twist and rise. This analysis yielded a twist of −53.91° and a rise of 19.03 Å, corresponding to a helical repeat unit composed of two copies each of CotV and CotW. We alternatively refined the reconstruction using a higher-order helical symmetry, characterized by a twist of 153.09° and a rise of 9.52 Å, which corresponds to one CotV–CotW heterodimer per asymmetric unit. This higher symmetry produced a markedly improved cryo-EM map, achieving a resolution of 3.32 Å.

An initial model was automatically built using ModelAngelo without the need for a preprovided protein sequence (22). Subsequently, detailed per-residue refinement of the initial model was performed using Phenix software package to produce the final refined model (27). Both the final model and initial Angelo model fit the cryo-EM map well, with most side-chain densities being accurately resolved and represented by the models (Fig. S1, Table S1).

The native ORFs of *B. subtilis* 168 encode CotV and CotW proteins comprising 129 and 105 amino acids, respectively. In our expression constructs, CotV was engineered with an N-terminal extension of 14 residues, including a 6×His tag for purification, and an additional C-terminal tyrosine residue for providing UV absorbance signal. CotW was expressed with a single C-terminal tyrosine extension. These engineered residues were not resolved in the cryo-EM density and were therefore excluded from the final atomic model. The refined structure includes residues 1 to 125 of CotV and 18 to 98 of CotW, corresponding to the well-ordered core regions of the heteromeric filament. The alignment between the full-length expressed proteins and the structurally resolved segments is depicted in Fig. S2*B*.

### Chemical and mechanical treatment for CotVW structural stability

One hundred microliters of CotVW complex was subjected to various treatments to evaluate its structural stability under chemical, physical, and stress-inducing conditions. The treatments included 8 M urea, 1% SDS with boiling, 1 M HCl, 1 M NaOH, heating at 80 °C for 20 min, sonication at 40% amplitude (10 s/cycle, triplicated), and incubation with 0.05 mg/ml subtilisin at 37 °C for 1 h. Following each treatment, the samples were examined using negative-stain EM to assess their structural integrity.

### Divalent metal ion treatment of CotVW filaments

A total of 1 ml of CotVW protein was incubated with 1 mM Ca^2+^, Mg^2+^, Mn^2+^, or Ni^2+^ for 20 min at room temperature. Following incubation, the samples were centrifuged at 21,000*g* for 20 min at 4 °C to separate the pellet and supernatant fractions. The pellet was then washed twice with pH 8.0 lysis buffer (20 mM Tris–HCl and 150 mM NaCl) containing 1 mM of the respective divalent metal ions, followed by additional centrifugation at 21,000*g* for 20 min at 4 °C after the final wash step. The pellet fraction was subsequently analyzed using negative-stain EM following the protocol described above.

### CotVW complex under varying pH conditions

Lysis buffers with different pH values were prepared, each containing 20 mM buffering agent and 150 mM NaCl. The specific pH conditions included Tris–HCl buffers at pH 8.0, and 7.0, Mes-NaOH buffer at pH 6.0, and acetate-NaOH buffer at pH 5.0 and 4.0. CotVW was purified using a pH-specific buffer, and the resulting samples were centrifuged at 21,000*g* for 20 min at 4 °C to separate the supernatant and pellet fractions. A comparative analysis of protein distribution in the supernatant and pellet fractions was performed using SDS-PAGE.

### Protein binding assay

SOD2 is a tetrameric protein, and 6xHis tagged SOD2 was used as a representative binding partner for coprecipitation with CotVW (28). The concentration of CotVW was adjusted using Bradford assays, and 50 μl of CotVW was mixed with 50 μl of SOD2-Mn protein (2 mg/ml) to obtain a total reaction volume of 100 μl. The mixtures were prepared at pH 8.0 and 6.0 conditions and incubated for 20 min at room temperature. Each protein was incubated separately under the same conditions as controls. Following incubation, the samples were centrifuged at 21,000*g* for 20 min at 4 °C to separate the supernatant and pellet fractions. The pellet was washed twice with the respective pH buffers (20 mM Tris–HCl, 150 mM NaCl at pH 8.0, 20 mM Mes-NaOH, and 150 mM NaCl at pH 6.0). After the final wash, the samples were centrifuged again at 21,000*g* for 20 min at 4 °C. A comparative analysis of the protein content in the final pellets was conducted using SDS-PAGE. A binding assay using a single CotY protein was performed following the same procedure.

## Data availability

The structures of the CotVW complex (PDB: 9LGH, whole map: EMD-63065) have been deposited to the Protein Data Bank (http://www.rcsb.org) and the Electron Microscopy Data Bank (https://www.ebi.ac.uk/emdb/), respectively.

## Supporting information

This article contains supporting information.

## Conflict of interest

The authors declare that they have no conflicts of interest with the contents of this article.
