# Supplementary material for: Filamentous structure of the CotVW complex, the crust proteins of the Bacillus subtilis endospore
Source: J Biol Chem. 2025 Sep 11;301(11):110714. doi: 10.1016/j.jbc.2025.110714 (PMC12630357; doi:10.1016/j.jbc.2025.110714)
Supplement: Supporting Figures and Table [file mmc1.pdf]

Filamentous structure of the CotVW complex, the crust proteins of the *Bacillus subtilis* endospore

Eunbyul Jo[1], Doyeon Kim[1], Yeongjin Baek[1], Migak Park[1], Hyojeong Lee[1], and Nam-Chul Ha[1,2*]

[1]Research Institute of Agriculture and Life Sciences, Department of Agricultural Biotechnology, CALS, Seoul National University, Seoul 08826, Republic of Korea

[2]Center for Food and Bioconvergence, Seoul National University, Seoul 08826, Republic of Korea

[*]To whom correspondence should be addressed: hanc210@snu.ac.kr

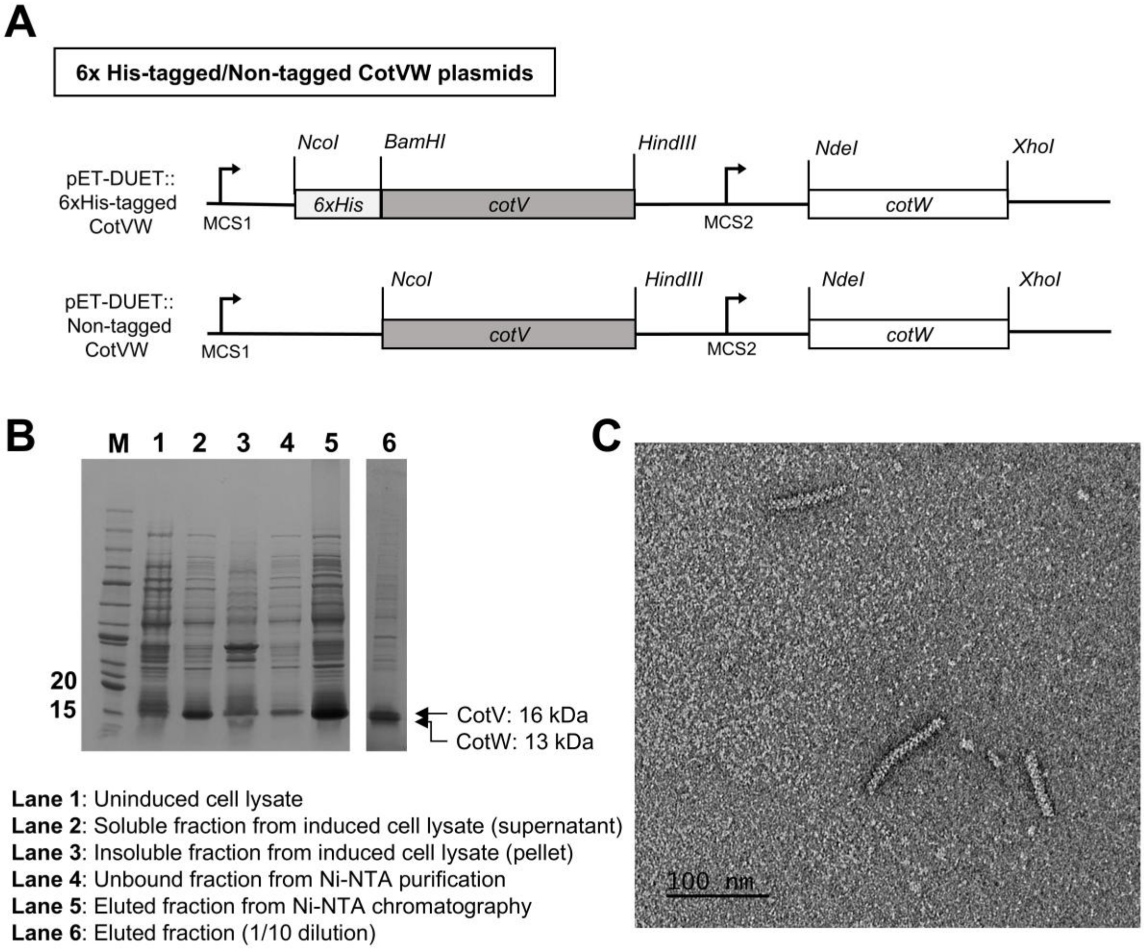

Supplementary Figure 1. Plasmid construct and expression of CotV and CotW.

A. Schematic representation of the pET-DUET::6×His-tageed, pET-DUET::Non-tagged CotVW construct. Enzyme restriction sites and promoter regions are labeled.

B. SDS-PAGE analysis of the CotVW complex during induction and Ni-NTA chromatography purification. A brief explanation of each lane is provided below the gel.

C. Negative-stain EM analysis of the eluted fraction from a Ni-NTA chromatographic column.

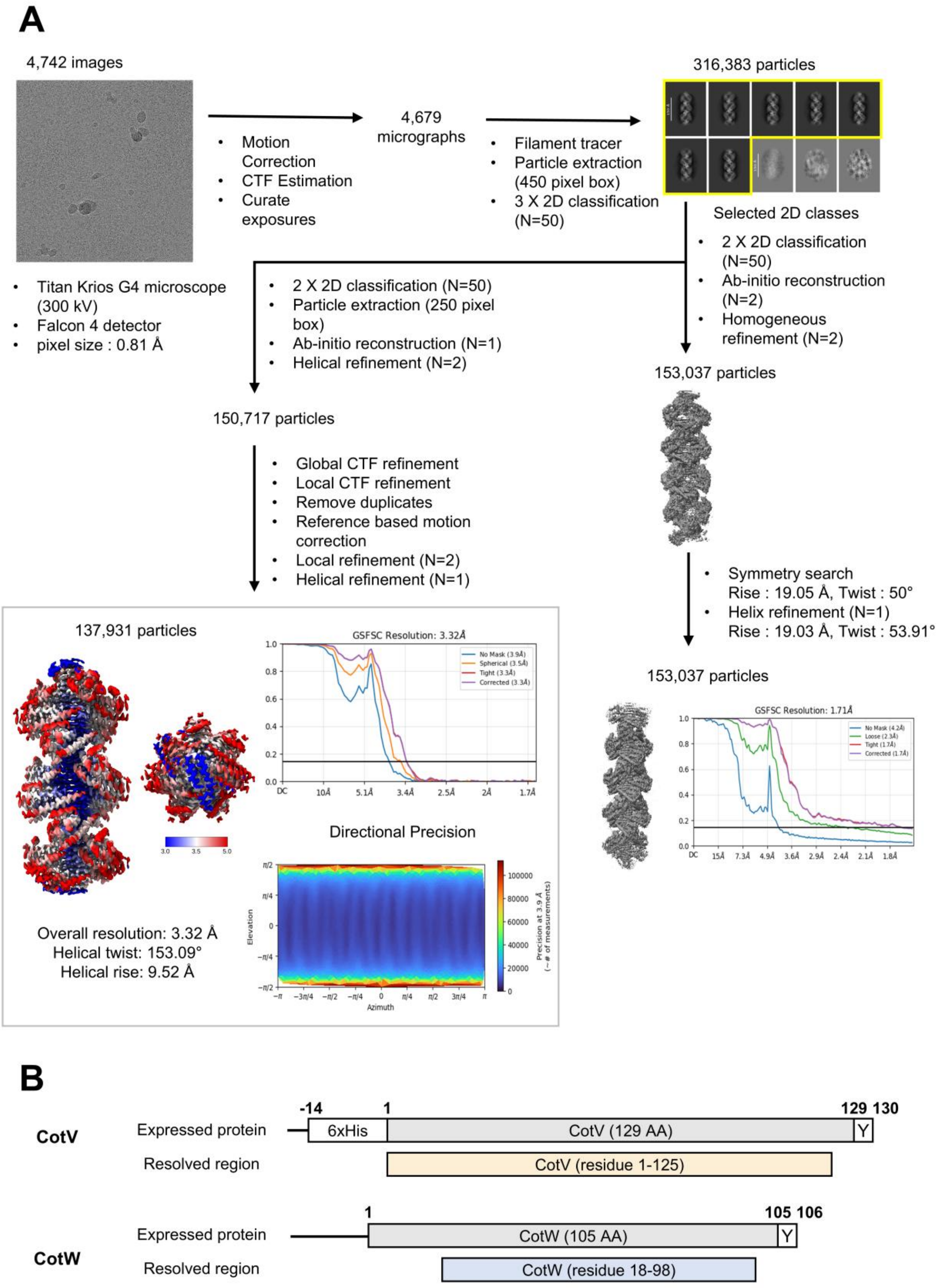
A
4,742 images
Motion Correction
CTF Estimation
Curate exposures
4,679 micrographs
Filament tracer
Particle extraction (450 pixel box)
3 X 2D classification (N=50)
316,383 particles
Selected 2D classes
Titan Krios G4 microscope (300 kV)
Falcon 4 detector
pixel size : 0.81 Å
2 X 2D classification (N=50)
Particle extraction (250 pixel box)
Ab-initio reconstruction (N=1)
Helical refinement (N=2)
2 X 2D classification (N=50)
Ab-initio reconstruction (N=2)
Homogeneous refinement (N=2)
153,037 particles
150,717 particles
Global CTF refinement
Local CTF refinement
Remove duplicates
Reference based motion correction
Local refinement (N=2)
Helical refinement (N=1)
Symmetry search
Rise : 19.05 Å, Twist : 50°
Helix refinement (N=1)
Rise : 19.03 Å, Twist : 53.91°
137,931 particles
GSFSC Resolution: 3.32Å
No Mask (3.9Å)
Spherical (3.5Å)
Tight (3.3Å)
Corrected (3.3Å)
DC
10Å
5.1Å
3.4Å
2.5Å
2Å
1.7Å
3.0
3.5
5.0
Directional Precision
Elevation
Azimuth
Precision at 3.9 Å (~ # of measurements)
Overall resolution: 3.32 Å
Helical twist: 153.09°
Helical rise: 9.52 Å
153,037 particles
GSFSC Resolution: 1.71Å
No Mask (4.2Å)
Loose (2.3Å)
Tight (1.7Å)
Corrected (1.7Å)
DC
15Å
7.3Å
4.9Å
3.6Å
2.9Å
2.4Å
2.1Å
1.8Å
B
CotV
Expressed protein
-14
1
129 130
6xHis
CotV (129 AA)
Y
Resolved region
CotV (residue 1-125)
CotW
Expressed protein
1
105 106
CotW (105 AA)
Y
Resolved region
CotW (residue 18-98)

Supplemental Figure 2. Cryo-EM processing workflow and model coverage for the CotVW filament.

A. Schematic workflow for the cryo-EM structure determination of the CotVW filament. Data were collected using a Titan Krios G4 microscope (300 kV) equipped with a Falcon 4 detector at a pixel size of 0.81 Å and acquiring 4,742 movies. Motion correction and CTF estimation were then performed using CryoSPARC (Patch Motion correction and Patch CTF estimation). Filament particles were automatically picked using the Filament Tracer tool, resulting in the extraction of 602,002 particles (box size: 450 pixels), which were subjected to three rounds of 2D classification. Further extraction (250 pixels) from 3,594 micrographs yielded 150,717 particles, which were refined to 137,931 particles for helical reconstruction. Helical refinement was conducted with an initial twist of 153.11° and rise of 9.515 Å, followed by a helical symmetry search within a range of 152.5°–154° twist and a 9–10 Å rise. Subsequent post-processing and helical refinement produced a final cryo-EM map at 3.32 Å resolution with a helical twist of 153.09° and helical rise of 9.52 Å. The final atomic model was constructed using the ModelAngelo and Phenix software.

B. Schematic representation of the expressed constructs and the resolved regions of CotV and CotW. Gray bars denote the full-length native sequences of CotV (129 residues) and CotW (105 residues) from *B. subtilis* 168. The "Expressed protein" constructs include an N-terminal 6×His-tag (CotV) and C-terminal tyrosine residues (both CotV and CotW), which were added for purification. These tag regions were not resolved in the cryo-EM map. Colored bars represent the residues included in the final atomic model: CotV (residues 1–125, light brown) and CotW (residues 18–98, light blue).

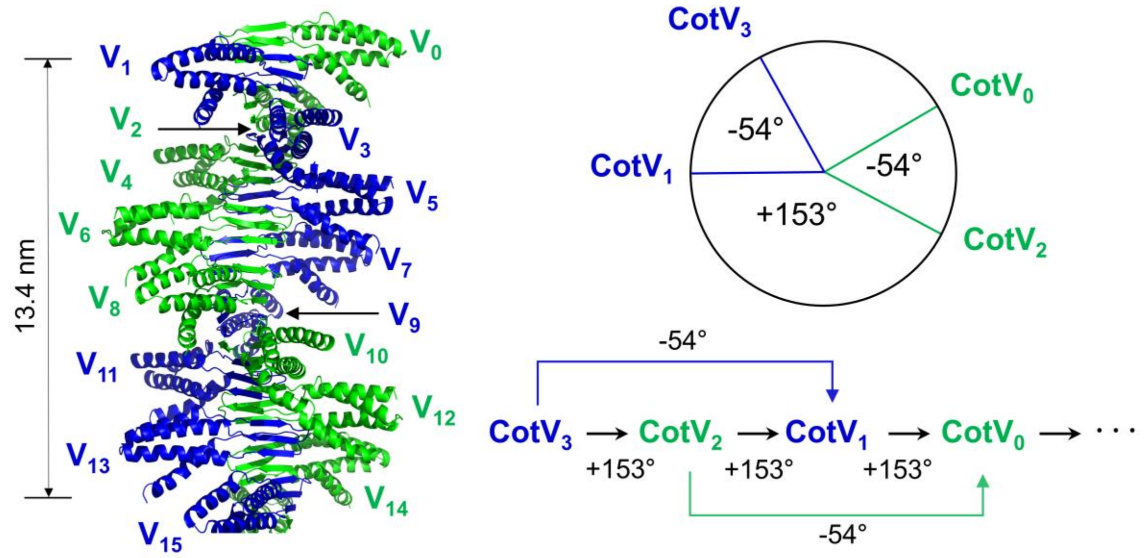

Supplemental Figure 3. Helical organization of the CotV filament.

The filament structure of CotV is shown, with the subunits labeled sequentially as V_0, V_1, V_2, V_3, … etc., from top to bottom. The subunits in odd number (V_1, V_3, V_5, etc.) are colored blue, whereas those in even numbers (V_0, V_2, V_4, etc.) are colored green. The right panel shows a top-down projection illustrating the relative torsion angles of the selected subunits ($CotV_0$, $CotV_1$, $CotV_2$, and $CotV_3$). The helical twist of +153° between directly adjacent subunits results in a -54° angle between every other subunit. The diagram below outlines the sequential rotational arrangement along the filament axis.

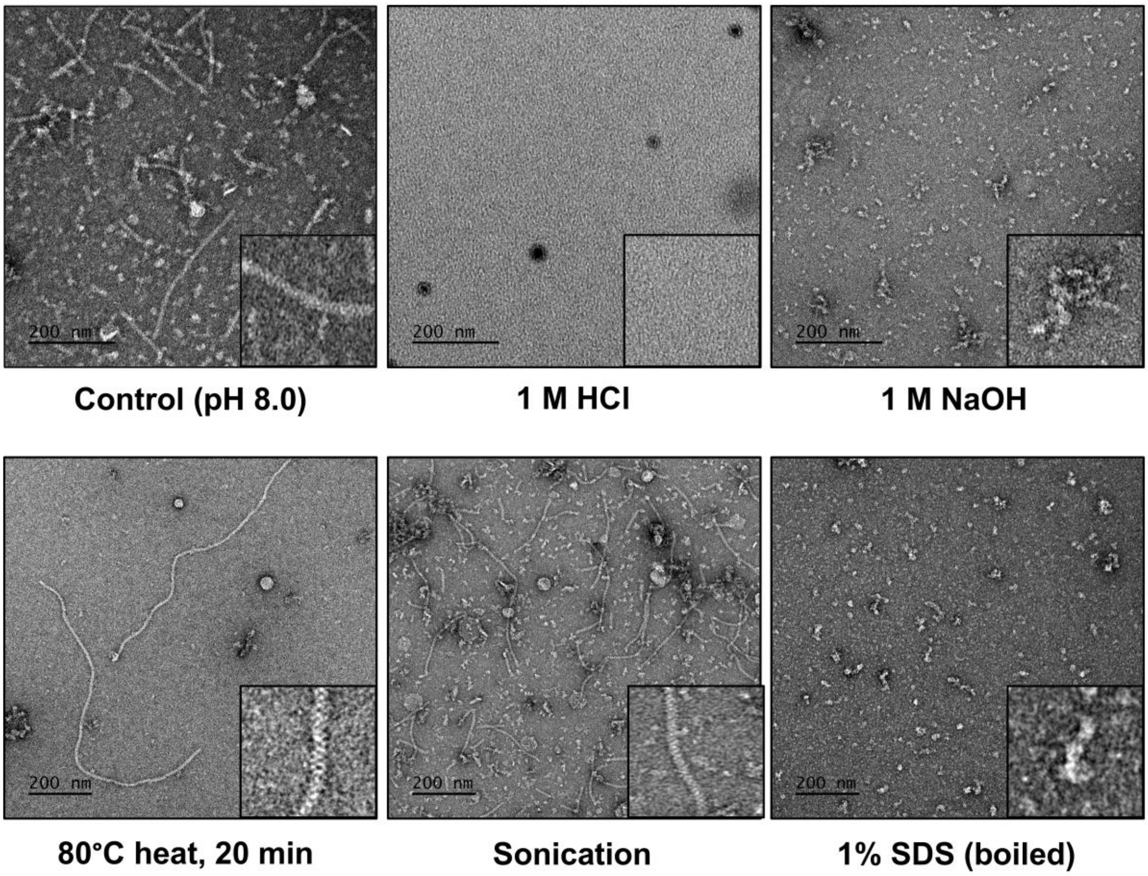

Supplemental Figure 4. Stability test of CotVW filaments under various stress conditions.

Negative-stain EM analysis of CotVW filaments after exposure to acidic, basic, heat (80°C), sonication, and 1% SDS with boiling. The filaments were subjected to each condition for 20 min, as indicated by the corresponding negative-stain EM images. The control condition corresponds to pH 8.0 (20 mM Tris-HCl, 150 mM NaCl).

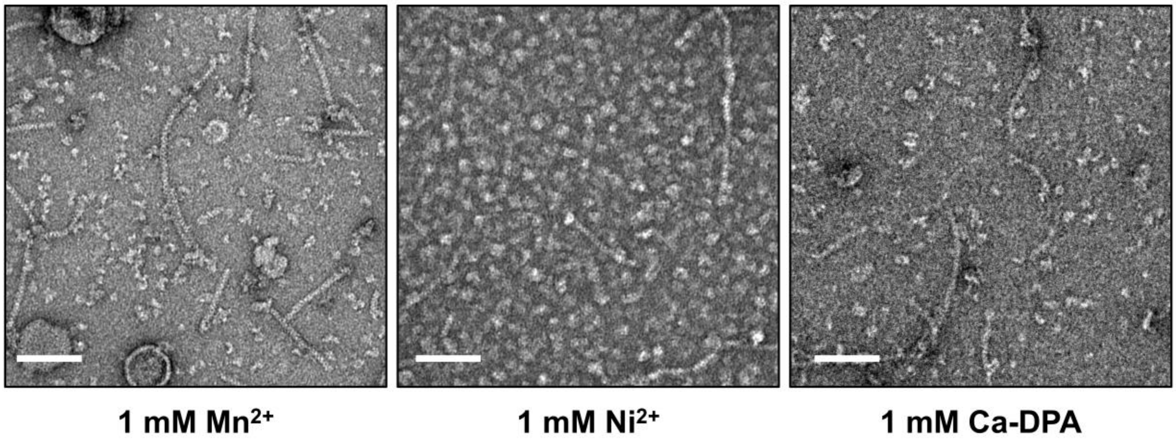

Supplemental Figure 5. Structural response of CotVW filaments to divalent cations.

Negative-stain EM images of Cot VW filaments after exposure to 1 mM $MnCl_2$, $NiCl_2$ or Ca-DPA for 20 min.

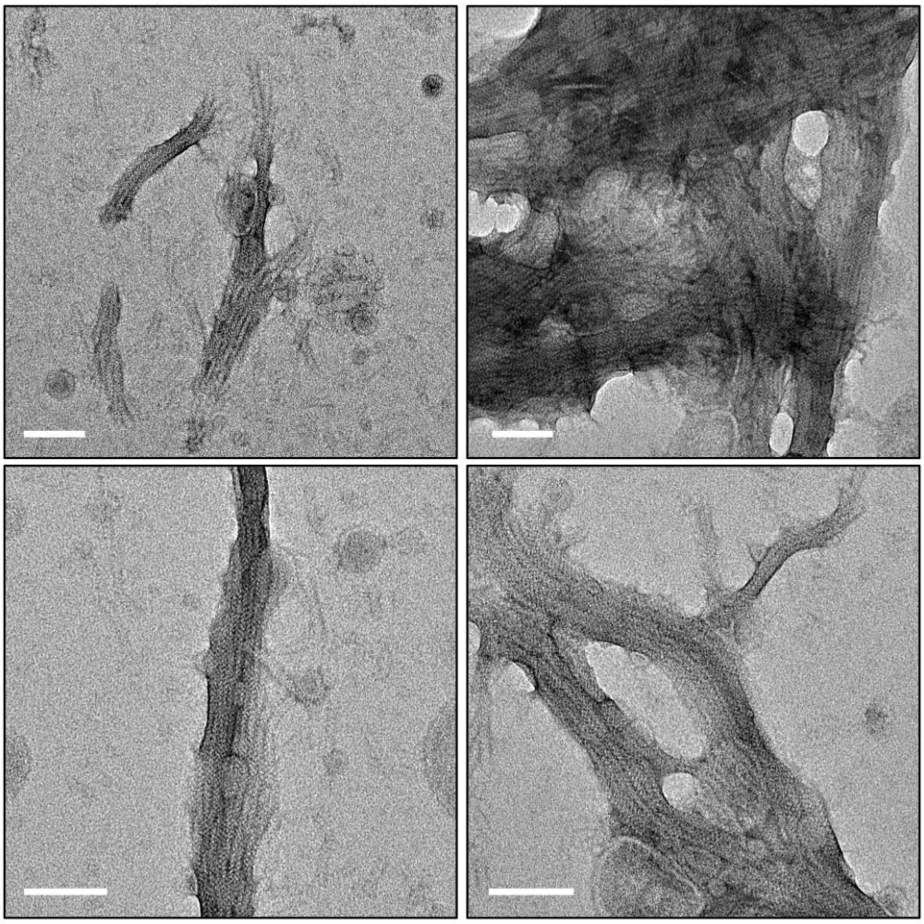

Supplementary Figure 6. pH-dependent bundling of CotVW filaments.

Negative-stain EM images of 6×His-tagged CotVW at pH 6.0, showing consistent filament bundling. Scale bars: 100 nm.

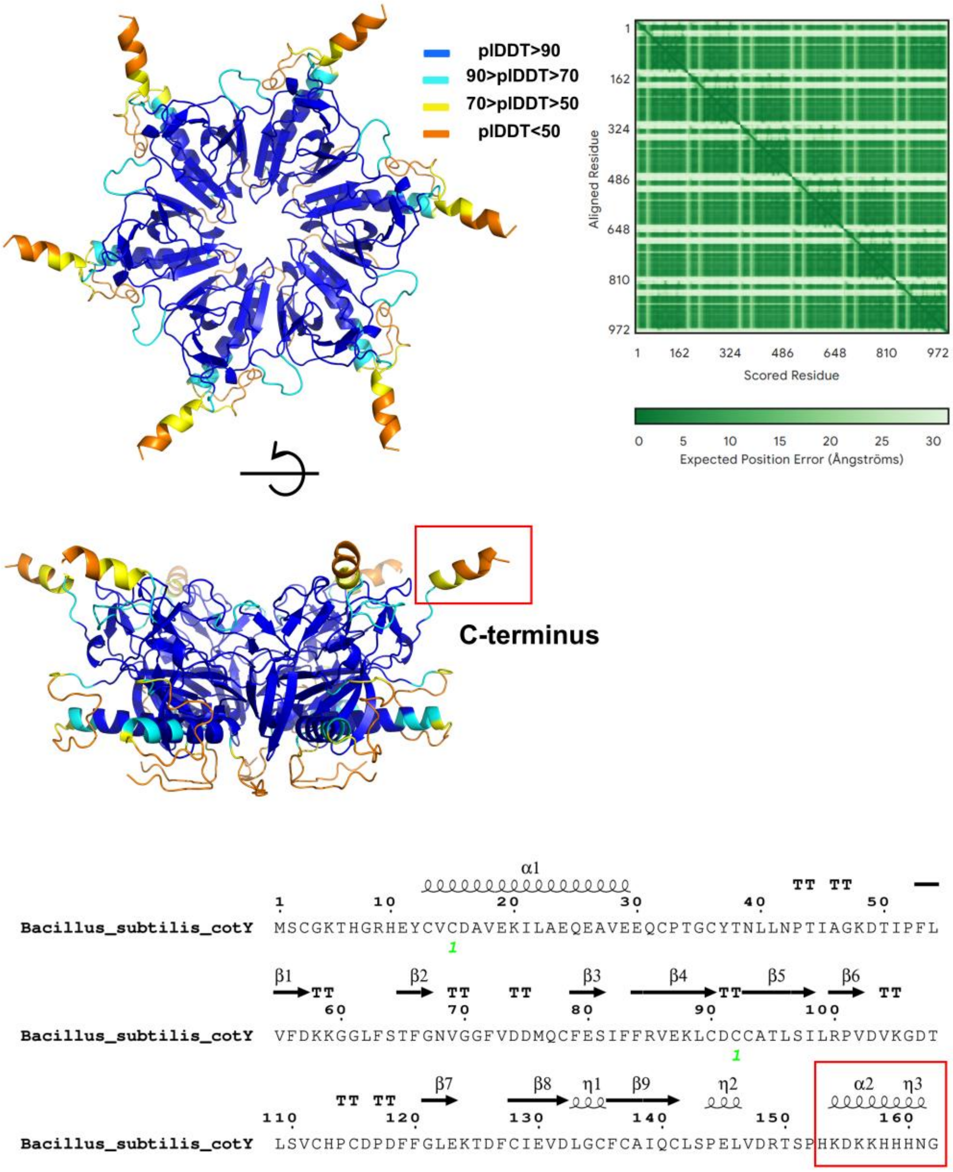

Supplemental Figure 7. AlphaFold3-predicted structure of *B. subtilis* CotY.

AlphaFold 3 predicted the structure of *B. subtilis* CotY in hexametric form. The right panel shows the plDDT graphs of the predicted structure. The C-terminus is highlighted with red rectangles.

Supplemental Table S1. Cryo-EM data collection, refinement, and validation statistics.

**Data collection and processing**	
Microscope	TFS Krios G4
Voltage (kV)	300
Camera	Falcon 4
Magnification	96,000
Pixel size (Å)	0.81
Defocus range (μm)	-1.7, -1.5, -1.3, -1.2, -1.1, -1.0, -0.9, -0.8
Total dose (e/Å²)	50
Dose per fraction (e/Å²)	2.08
C2 lens aperture	70
Total images	4,742
Initial number of particles	602,002
Final number of particles	137,931
Symmetry imposed	Helical/C1
Rise/twist	9.52 Å / +153.09°
Map resolution (Å)	3.32
FSC threshold	0.143
Local resolution range	3.0–5.0
Refinement; Initial model used	NA
**Ramachandran plot**	
Favored (%)	95.54
Allowed (%)	4.46
Disallowed (%)	0
**Validation**	
Clashscore	5
Ramachandran outliers	0
Side-chain outliers (%)	2.6
PDB ID	9LGH
EMDB ID	EMD-63065
